# Recent advances in endovascular treatment of peripheral arterial disease

**DOI:** 10.12688/f1000research.20398.1

**Published:** 2020-02-18

**Authors:** Andrew Lazar, Nicholas Morrissey

**Affiliations:** 1 Division of Vascular Surgery, Columbia University Irving Medical Center, New York, NY, 10032, USA

**Keywords:** Peripheral Arterial Disease, Endovascular Therapy, Classification Systems

## Abstract

As associated co-morbidities have transformed over time, the evaluation and management of peripheral arterial disease have evolved as well. New classification systems have been created to better understand the severity of a patient’s condition and the risk of amputation. These classifications include the Wound, Ischemia, and Foot Infection (WIfI) and Global Anatomic Staging System (GLASS) classification systems. Through the utility of these systems, a patient’s disease can be appropriately staged and managed with medical, endovascular, or surgical therapies or a combination of these. Endovascular therapies specifically have grown with the explosion of new technologies. There are numerous options for patients with disease amenable to endovascular therapy. In this review article, we discuss a number of these different endovascular therapies as well as the new classification systems.

Peripheral arterial disease (PAD) has changed over time as the prevalence of associated risk factors and co-morbidities—including the increase in insulin-dependent diabetes and the changing dynamic of tobacco use—has evolved ^1^. As the paradigm shifts on the basis of disease presentation, it has required improved classification systems. In 2014, the Society for Vascular Surgery presented the Wound, Ischemia, and Foot Infection (WIfI) classification system for threatened lower extremities ^2^. This system provides a more in-depth framework for the evaluation of patients with ischemia of the lower extremity and provides some guidance for the risk of limb loss associated with their condition. This classification system has four grades of severity for each of the following: wound, ischemia, and presence of infection. The wound grade is based on the presence of gangrene or ulcers (or both). The ischemia grade is based on ankle-brachial index, arterial systemic pressure, and toe pressures. The infection grade is based on signs and symptoms of infection. Subsequently, the Society for Vascular Surgery, the European Society for Vascular Surgery, and the World Federation of Vascular Societies created the “Global Vascular Guidelines (GVGs) on the management of chronic limb-threatening ischemia” ^1^. In these guidelines, they recommended a transition in terminology from “critical limb ischemia” to the more descriptive term of chronic limb-threatening ischemia, which objectively includes patients with ischemic rest pain or tissue loss due to atherosclerotic arterial disease. Additionally, they outlined a method of classifying patients by using anatomic (Global Limb Anatomic Staging System, or GLASS) and limb risk (WIfI classification) staging of the threatened lower extremity ^1^. The anatomic and limb risk staging combine to provide a recommendation treatment including “no revascularization”, “endovascular”, “indeterminate”, or “open bypass”. It is hoped that, through these new criteria, the GVGs provide clarity in the complex decision making of endovascular intervention or open surgery.

Although in years past open surgery was the gold standard for lower extremity revascularization, the advent of new technologies and devices has improved the outcomes with endovascular therapy. With so many different endovascular treatment modalities available, we provide a brief background on each type and some of the benefits and disadvantages of each. Although the focus of this review article is on endovascular therapies, one should remember that these described interventions are not the only, or potentially even the first-line, interventions. Aggressive medical and procedural therapies using a team-based approach have proven to be extremely important in limb preservation ^3, 4^. Additionally, there continues to be controversy among vascular specialists regarding whether endovascular or open surgical treatment should be first. The Best Endovascular versus Best Surgical Therapy for Patients With Critical Limb Ischemia (BEST-CLI) trial is currently investigating this question via a randomized controlled trial ^5^. Although the decision whether to proceed with endovascular or open surgical therapy first remains a complicated one without a clear answer, the remainder of this review will focus strictly on endovascular therapies.

## Percutaneous transluminal angioplasty

Following diagnostic angiography, one of the mainstays of endovascular therapy is percutaneous transluminal angioplasty (PTA). This involves placing a wire intra-luminally beyond the target lesion and then expanding the inserted balloon at the lesion to the appropriate pressure which leads to fracture of the lesion and stretching of the arterial wall. Standard angioplasty balloons have nominal and burst pressures. Nominal pressure is defined by expansion to the defined size, whereas burst pressure is when less than 1% of balloons would rupture ^6^. With device advances, a number of angioplasty balloons beyond the standard balloon are available. These include cutting balloons (CBs), cryoplasty balloons, focal pressure balloons, and drug-coated balloons (DCBs).

CBs have a number of longitudinal microsurgical blades that create multiple atherotomies upon expansion. Usually, CBs are used for short segment compatible disease, including stenosis of a bypass graft, in-stent restenosis, bifurcation stenosis (profunda/superficial femoral artery or tibioperoneal), and areas not traditionally amenable to stents (common femoral and popliteal arteries). Although CB angioplasty did not reduce recurrent in-stent restenosis, the Restenosis Cutting Balloon Evaluation Trial (RESCUT) noted that there were procedural advantages, including less balloon movement and use of fewer balloons ^7^.

Cryoplasty balloons function by combining hypothermia and pressure. The combination, in theory, helps to induce an inflammatory response and dilate the plaque. Performing cryoplasty does require additional technology to inflate the balloon with liquid nitrous oxide and thus entails an increased cost. The utility of this technology has not yet been proven to have improved outcomes or benefits over traditional PTA ^6^.

DCBs evolved from drug-eluting stents (DESs) which took advantage of the inhibition of intimal hyperplasia due to local medication administration. Initially, a standard PTA is performed and this is followed by deployment of the DCB. One of the broadly used medications on DCBs is paclitaxel, which is a commonly used chemotherapeutic. DCBs have been shown to have outcomes (target lesion revascularization and freedom from restenosis) superior to those of standard PTA without difference in amputation rate ^8, 9^. However, in December 2018, Katsanos *et al*. published a meta-analysis demonstrating an increased long-term mortality in the paclitaxel-coated balloon group ^10^. These results caused the US Food and Drug Administration to recommend, “when making treatment recommendations, and as part of the informed consent process, consider that there may be an increased rate of long-term mortality in patients treated with paclitaxel-coated balloons and paclitaxel-eluting stents,” ^11^ but it has since allowed previously paused randomized controlled trials to restart to study the topic in August 2019.

Recent technological advances have brought about newer angioplasty balloons engineered to disseminate the force on the vessel wall with less overall trauma. One such technology is the Chocolate PTA Balloon (TriReme Medical LLC, Pleasanton, CA, USA), which has a nitinol scaffold that reduces the overall balloon into subsegments. This provides increased overall surface area of the balloon and in prospective studies has been found to have low rates of complication, including dissection with improved 12-month primary patency of 64.1% ^12^.

## Endovascular stents

Initially, stents were made of a composition of metals to form a tube that could be placed intra-luminally. Stents have evolved to include bare metal stents (BMSs), covered stents, and DESs. They can also be balloon-expandable and self-expanding. Currently, stents are predominantly composed of nitinol, nickel and titanium, stainless steel, or cobalt chromium ^6^.

Balloon-expandable stents are mounted onto a delivery system over an angioplasty balloon. These devices are rigid to prevent movement and allow for precise placement however, balloon-expandable stents are at risk of becoming deformed because of external compression. Often, these can be used in iliac, subclavian, mesenteric, and renal arteries. These stents are often composed of stainless steel or cobalt chromium.

Self-expanding stents (SESs) are mounted alone on a device, and the radial force of the material causes the stent to expand outward upon deployment. SESs are usually made of nitinol, which provides the flexibility and memory that make up the stents’ primary function of exerting an expansile force on the diseased vessel wall. This force is paramount and thus when selecting an SES one should select a size larger than the vessel diameter. The properties of nitinol also allow the SES to be a useful device for tortuous and more difficult vessels to navigate.

Balloon and self-expanding stents are also available as covered-stent grafts. In the Covered versus Balloon Expandable Stent Trial (COBEST), covered stents were compared with BMSs in aorto-iliac disease. Covered stents were noted to demonstrate improved patency at 5 years, and fewer procedures for revascularization were required for TransAtlantic Inter-Society Consensus (TASC) C and D lesions ^13^. Although Piazza *et al*. found comparable results in iliac arteries between BMSs and covered stents, they also noted a significant improvement in patency in TASC D lesions with covered stents ^14^.

Also available are DESs, which are similar to DCBs and which were created in an attempt to overcome stent thrombosis and in-stent restenosis. Like DCBs, the stents are coated in agents, including paclitaxel and sirolimus. The DES was born out of the coronary intervention repertoire; however, it has been applied to peripheral interventions as well. Although it has not demonstrated an improvement in overall amputation, the DES was shown to have superiority to the BMS in PAD with less restenosis and improved target revascularization ^8^. With the DES, as with the DCB, concern over the impact of paclitaxel on mortality has been raised and requires further investigation.

Lastly, bioabsorbable stents have been developed using biodegradable polymers. Although there is promise in the idea of a bioabsorbable material, this has not been shown in randomized controlled trials and has not demonstrated improved or equal patency compared with endarterectomy and PTA. From these results, further research and development are required to enhance the outcomes over more standardized interventions ^15, 16^.

## Atherectomy

Although balloon angioplasty and stenting have provided a strong base for endovascular therapy, atherectomy developed as an additional option in anatomic locations not normally amenable to stents, including the common femoral artery or popliteal artery. Atherectomy has become widely adopted across specialties with four main methods of function: directional, rotational, orbital, and laser atherectomy.

Directional atherectomy functions using a carbide cutting blade with varying sizes to customize to the type of lesion. A distal protection device is placed and then the blade rotates and is moved across the lesion. As the blade is advanced, the particles are contained by the protection device ^6, 17^.

Rotational atherectomy uses a metallic drill that, with high-speed rotation, can be used on a variety of different plaques, including calcified and soft thrombus. Specific devices are also accompanied by an aspiration system to prevent distal embolization, although the fragments of plaque are predominantly under 5 microns ^6^.

Orbital atherectomy is similar to rotational atherectomy and uses an abrasive diamond-coated crown that rotates to grind debris down to particles small enough not to cause problematic distal embolization. This technique was studied in the CALCIUM 360 Pilot trial in which PTA appeared to be more successful after orbital atherectomy ^18^. However, this was a small pilot trial with 50 patients and short follow-up. Of note, this device appeared to be most useful on heavily calcified lesions.

Lastly, laser atherectomy uses highly focused light to ablate lesions by direct contact with minimal surrounding thermal damage. This technique with PTA demonstrated superiority over standard PTA in the Excimer Laser Randomized Controlled Study for Treatment of Femoropopliteal In-Stent Restenosis (EXCITE ISR) showing significant procedural success with a decrease in major adverse events ^19^.

Although atherectomy has been adopted widely, large numbers of randomized controlled trials studying atherectomy techniques against PTA are still lacking. Additional confounders include the best anatomic location and type of plaque that atherectomy should be used for. With this in mind, it is apparent that significant further study is necessary to evaluate the most appropriate indication for atherectomy.

## Lithoplasty

Among other new technologies currently under investigation is the Shockwave Lithoplasty System (Shockwave Medical, Fremont, CA, USA), which adapts technology used in the treatment of renal calculi. This technology uses a catheter-guided balloon that produces powerful acoustic shockwaves to disrupt plaque while reducing vessel wall injury. The technology is being studied in the DISRUPT PAD III clinical trial ^20^.

## Conclusions

The growth of PAD and its associated complications has led to improved recognition and creation of useful classifications by vascular societies, including the WIfI and GLASS classification systems. Additionally, as limb preservation continues to be the goal for patients with PAD, endovascular interventions have played a larger role. Although standard PTA and stent placement demonstrated good patency, there is a push to create technology with improved outcomes. A number of available technologies have been discussed and many others are on the horizon. Although some devices have demonstrated superiority, it is clear that significant research and clinical trials are necessary to determine the specific utility and effectiveness of each device.
